# Targeting Rapamycin to Podocytes Using a Vascular Cell Adhesion Molecule-1 (VCAM-1)-Harnessed SAINT-Based Lipid Carrier System

**DOI:** 10.1371/journal.pone.0138870

**Published:** 2015-09-25

**Authors:** Ganesh Ram R. Visweswaran, Shima Gholizadeh, Marcel H. J. Ruiters, Grietje Molema, Robbert J. Kok, Jan. A. A. M. Kamps

**Affiliations:** 1 Department of Pathology & Medical Biology, Medical Biology Section, University Medical Center Groningen, University of Groningen, Groningen, the Netherlands; 2 Department of Pharmaceutics, Utrecht Institute for Pharmaceutical Sciences, Utrecht University, Utrecht, the Netherlands; Wuhan University of Science and Technology, CHINA

## Abstract

Together with mesangial cells, glomerular endothelial cells and the basement membrane, podocytes constitute the glomerular filtration barrier (GFB) of the kidney. Podocytes play a pivotal role in the progression of various kidney-related diseases such as glomerular sclerosis and glomerulonephritis that finally lead to chronic end-stage renal disease. During podocytopathies, the slit-diaphragm connecting the adjacent podocytes are detached leading to severe loss of proteins in the urine. The pathophysiology of podocytopathies makes podocytes a potential and challenging target for nanomedicine development, though there is a lack of known molecular targets for cell selective drug delivery. To identify VCAM-1 as a cell-surface receptor that is suitable for binding and internalization of nanomedicine carrier systems by podocytes, we investigated its expression in the immortalized podocyte cell lines AB8/13 and MPC-5, and in primary podocytes. Gene and protein expression analyses revealed that VCAM-1 expression is increased by podocytes upon TNFα-activation for up to 24 h. This was paralleled by anti-VCAM-1 antibody binding to the TNFα-activated cells, which can be employed as a ligand to facilitate the uptake of nanocarriers under inflammatory conditions. Hence, we next explored the possibilities of using VCAM-1 as a cell-surface receptor to deliver the potent immunosuppressant rapamycin to TNFα-activated podocytes using the lipid-based nanocarrier system Saint-O-Somes. Anti-VCAM-1-rapamycin-SAINT-O-Somes more effectively inhibited the cell migration of AB8/13 cells than free rapamycin and non-targeted rapamycin-SAINT-O-Somes indicating the potential of VCAM-1 targeted drug delivery to podocytes.

## Introduction

Kidney glomeruli are composed of four major components namely mesangial cells, fenestrated endothelium, glomerular basement membrane (GBM), and podocytes. These latter cells form the glomerular filtration barrier (GFB) of the kidney. Mesangial cells, present in the interstitium between the glomerular endothelial cells, are indirectly involved in the filtration process by controlling the glomerular surface area [1]. The glomerular endothelium is lined with 70–100 nm fenestrations which are actively involved in filtration [1]. The fenestrated endothelium is attached to one side of the GBM, which contain pores of around 250–350 nm. The GBM is sandwiched on the proximal side by visceral epithelial cells called podocytes. The adjacent podocytes are connected by slit diaphragms with a width of around 40 nm, forming podocyte foot processes mainly involved in filtration of proteins [1]. The slit diaphragm bridging the podocytes contains nephrin, podocin, CD2AP, and nephrin-like proteins NEPH1 and NEPH2. Contraction and relaxation of the podocytes regulate the glomerular filtration rate leading to ultrafiltration of water and ion salts into the urinary space. Apart from filtration, podocytes synthesize the GBM, maintain the shape of the capillary tube, and provide growth factors for mesangial and endothelial cells. Recent findings demonstrate that cross-talk exists between the endothelial cells and podocytes, especially during glomerulopathies, indicating a significant role of these cells in glomerular disease [2].

Glomerulopathies in the USA comprise 90% of patients that need kidney transplantation or dialysis emphasizing the importance of the development of efficacious drugs to treat injured glomerular cells [3]. In glomerulopathies such as glomerular nephritis, glomerular sclerosis and diabetic nephropathy, especially the podocytes are severely affected leading to partial or complete loss of filtration function [1]. During these diseases, the fenestrations of the glomerular filtration barrier widen, leading to proteinuria, the hallmark of glomerular diseases. Further, detachment of the podocyte foot-processes and subsequent loss of the podocytes in urine dramatically increases proteinuria. To date, therapeutic intervention to treat podocytopathies is inadequate. Because of their role in pathological processes of glomerular diseases and their accessibility through the blood stream of glomerular capillaries, podocytes could be targeted by systemically applied drug-targeting approaches employing molecular targets specifically expressed by the diseased podocytes. Although there are few preliminary studies involving delivery of siRNA and proteins to podocytes [4,5], targeting small molecule therapeutics was never done. Rapamycin (Sirolimus), a mammalian target of rapamycin (mTOR) inhibitor, is widely used in the clinic as immunosuppressant to treat patients after kidney transplantation. However, the prolonged use of rapamycin in kidney transplant patients results in severe proteinuria that leads to the progression of chronic kidney disease and renal failure [6]. Furthermore, it has been shown that mTOR complex 1(mTORC1) is involved in podocyte injury [7]. Using targeted drug delivery systems such as immunoliposomes that accumulate selectively in designated target cell types, the dosage of rapamycin may potentially be optimized. The selective delivery of rapamycin in podocytes can minimize the total dose needed and can minimize the exposure of other cells to this potent mTOR inhibitor, thus potentially reducing the risk for serious side effects.

In this study, we investigated whether podocytes express inflammation-induced cell adhesion molecules like vascular cell adhesion molecule-1 (VCAM-1). VCAM-1 is a surface-expressed receptor that can facilitate receptor-mediated endocytosis of nanosized drug carriers. We used lipid-based nanocarriers called SAINT-O-Somes that were previously shown to have better intracellular drug release properties than conventional liposomes, and that can target a particular cell type when harnessed with an antigen-specific antibody [8,9]. Here, we report that cultured podocytes express VCAM-1 when triggered with TNFα to mimic the inflammatory condition. Furthermore, as a conceptual proof of targeting drugs to podocytes, we demonstrated that these cells internalize this type of lipid-based nanocarriers and, finally, that rapamycin incorporated into VCAM-1-SAINT-O-Somes effectively inhibited cellular responses in VCAM-1-expressing podocytes.

## Materials and Methods

### Materials

Lipids 1,2-distearoyl-*sn*-glycero-3-phosphoethanolamine-N-[methoxy(polyethylene glycol)-2000]-maleimide (Mal-PEG_2000_-DSPE), 1-palmitoyl-2-oleoyl-*sn*-glycero-3-phosphocholine (POPC) and 2-distearoyl-*sn*-glycero-3-phosphoethanolamine-N-[methoxy(polyethylene glycol)-2000] (DSPE-PEG_2000_) were obtained from Avanti Polar Lipids (Alabaster AL, USA). Cholesterol (Chol) and N-succinimidyl-S-acetylthioacetate (SATA) were purchased from Sigma-Aldrich (St. Louis MO, USA). 1,1′-dioctadecyl-3,3,3′,3′-tetramethylindocarbocyanine perchlorate (DiI) was from Molecular Probes (Leiden, the Netherlands) and 1-methyl-4-(cis-9-dioleyl)methyl-pyridinium-chloride (SAINT-C18) from Synvolux Therapeutics Inc. (Groningen, the Netherlands). All chemicals used were of analytical grade unless otherwise stated.

### Cell Culture

The generation of the human and mouse conditionally immortalized podocyte cell lines, AB8/13 and MPC-5, were previously described [10,11]. AB8/13 podocytes were kindly provided by Dr. Moin A. Saleem (Bristol, UK); Dr. Peter Mundel (Charlestown, USA) kindly provided the MPC-5 cells. AB8/13 and MPC-5 cells were cultured at 33°C and the cells were differentiated in 5% CO_2_ incubator at 37°C for 10–15 days as indicated. The cells were cultured in RPMI 1640 medium containing 1% 100 units/ml penicillin/streptomycin and 1% Insulin-Transferrin-Selenium (ITS) (Invitrogen, Breda, the Netherlands) and 10% fetal bovine serum. For MPC-5 cells, ITS was excluded and pyruvate (1%) and interferon (10 U IFN-γ/ml) were added for cell proliferation. For differentiation of MPC-5 cells, medium without IFN-γ was used and the cells were incubated at 37°C in collagen A (10%)-coated cell culture plates (BD Biosciences, Breda, the Netherlands). The medium was refreshed at 5-day intervals. On the day of the experiment, cells were incubated in the absence (quiescent cells) or presence of TNFα (Boehringer Ingelheim GmbH, Ingelheim am Rhein, Germany) at 10 ng/ml for 24 h, unless stated otherwise.

### Isolation of Primary Podocytes

Male C57bl/6OlaHsd mice (18–23 g) were purchased from Harlan (Zeist, The Netherlands). Animals were group housed and maintained on a mouse chow diet, in a temperature and light-dark cycle controlled environment (24°C, 12:12 h). Mice were sacrificed under anesthesia (inhalation of isoflurane/O_2_), and organs were collected. All animal experiments were performed according to national guidelines and upon approval of the local Animal Care and Use Committee of Groningen University (DEC 6106).

Glomeruli from kidneys of 6–8 C57BL/6 mice were isolated based on previously described anti-CD31bead based endothelial cell preparation [12] with the following modifications. Cortices of the kidneys were minced for 3 min and next incubated at 37°C with collagenase-I (0.2 g% in Dulbecco's phosphate buffered saline (DPBS) with Ca^2+/^Mg^2+^; Worthington Biochemical Corp. Lakewood, NJ, USA) for 45 min. The collagenase/kidney mixture was pushed 15–17 times through a 14 G needle and dripped gently through a 70 μm cut-off cell strainer (BD Pharmingen, San Diego, CA). The eluate was spun down at 140 x g for 8 min and the pellet was resuspended in ~3 ml of bead wash solution (PBS with 0.1% BSA and antibiotic/antimycotic (1:100) (Sigma) followed by incubation with rat anti mouse-CD31 (BD Pharmingen)-coated magnetic beads (pre-incubated overnight at 4°C with sheep anti-rat beads; (Dynabeads, Life Technologies, Bleiswijk, the Netherlands)) for 15 min at room temperature on a rotator. The anti-CD31 positive cells were separated using a Magnetic Particle Concentrator (Invitrogen) and the bead discard was sieved through a 40 μm cut-off cell strainer (BD Pharmingen) to isolate glomeruli. The glomeruli on cell strainer were washed with 25 ml of DPBS at 5 ml aliquots. The glomeruli were next treated with 1 ml of collagenase-I (Worthington Biochemical Corp.) in DPBS with Ca/Mg for 1 h followed by 10 times titruation using an insulin syringe. The glomeruli were obtained by spinning down at 140 x g for 8 min and resuspended in Dulbecco’s Modified Eagle Medium (DMEM)/F-12 supplemented with 10% FCS, 100 μg/ml streptomycin, 100 U/ml pencillin and Insulin-Transferrin-Selenium (ITS) (podocyte-specific medium) and seeded in gelatin-coated plates at 37°C for 4–5 days. To eliminate the endothelial cells from the glomerular outgrowths, anti-ICAM-2 (BD Pharmingen)-coated magnetic beads (pre-incubated overnight at 4°C with sheep anti-rat beads; Dynabeads) were incubated with trypsin/EDTA detached glomerular outgrowth cells for 15 min at room temperature on a rotator. The ICAM-2 positive cells were removed from the glomerular outgrowth cells and the ICAM-2 negative fraction was seeded in podocyte-specific medium until confluent (2–3 days) at 37°C and RNA was next isolated for RT-qPCR analysis. Cytospots of the ICAM-2 negative fraction cells were made on microscope slides for immunostaining by releasing the cells from the tissue culture plates by trypsinization followed by suspending the cells in podocyte-specific medium.

### Gene Expression Studies by RT-qPCR

RNA was isolated from cells using the RNeasy® Mini Plus Kit (Qiagen, Venlo, the Netherlands) according to manufacturer’s protocol. The concentration and integrity of RNA were determined by the NanoDrop® ND-1000 spectrophotometer (Wilmington, DE) and by agarose (1%) gel electrophoresis, respectively. Complementary DNA (cDNA) from respective RNA samples were generated using SuperScript™ III RNaseH-Reverse Transcriptase (Invitrogen), RNaseOut inhibitor (40 U) (Invitrogen) and 250 ng random hexamers (Promega, Leiden, the Netherlands) in 20 μl total volume. 1 μl of the cDNA product was used for each PCR reaction.

Human GAPDH (Hs99999905_m1), E-selectin (Hs00174057_m1), VCAM-1 (Hs00365486_m1), WT-1 (Hs01103751_m1) and mouse GAPDH (Mm99999915_g1), synaptopodin (Mm03413333_m1), WT-1 (Mm01337048_m1) and VCAM-1 (Mm00449197_m1) primer- probes were purchased as Assay-on-Demand from Applied Biosystems (Nieuwekerk a/d IJssel, the Netherlands). These primer-probes together with Absolute QPCR Rox Mix (Thermo Scientific, Landsmeer, the Netherlands) were used in Quantitative (q) PCR performed in ABI PRISM 7900HT Sequence Detector (Applied Biosystems), with two technical replicates per sample. RT-qPCR and data analysis were performed as described previously [9]. The threshold cycle values (C_t_) were obtained by the comparative C_t_ method and we averaged the two technical replicate C_t_ values. The respective genes were normalized to the housekeeping gene glyceraldehyde-3-phosphate dehydrogenase (GAPDH), resulting in ΔC_t_ values. The relative mRNA levels were calculated by 2^-ΔCt^ for respective genes.

### Western Blot

AB8/13 cells, differentiated for 15 days at 37°C, treated with TNFα (10 ng/ml) for 4, 8 and 24 h were lysed by sonication in Radio-Immuno Precipitation Assay (RIPA) buffer (Sigma-Aldrich) for 1 min. Total protein concentration of the lysate was determined by BCA protein assay (Thermo Scientific) according to manufacturer’s protocol and equal amounts of lysate were mixed with SDS-loading buffer. The protein samples were electrophoresed on a SDS-(10%) PAA gel and transferred to a nitrocellulose membrane followed by blocking with 5% skimmed milk in PBS for 1 h. The membrane was cut based on the molecular masses of the target proteins followed by incubation with primary antibodies rabbit- polyclonal anti-VCAM-1 (~100-kDa, Santa Cruz Biotechnology Inc. Heidelberg, Germany), mouse-anti-human synaptopodin (~74-kDa, Progen), rabbit-polyclonal anti-GAPDH (~37-kDa, Santa Cruz Biotechnology Inc.) and/or mouse anti-human actin (~42-kDa, Millipore, Amsterdam, the Netherlands) for 1 h at room temperature. The membranes were washed three times with PBST and incubated with relevant horseradish peroxidase (HRP)-conjugated secondary antibodies for 1 h. Finally, the membranes were washed and the proteins on the membrane were visualized and detected using Supersignal West Femto Chemiluminescent Substrate (Thermo Scientific) and a Gel Doc imaging system equipped with a XRS camera (Bio-Rad).

### SAINT-O-Somes Preparation

DiI-Saint-O-Somes (SAINT-O-Somes) were prepared from Mal-PEG_2000_-DSPE, DSPE-PEG_2000_, SAINT-C-18, POPC and cholesterol in a molar ratio of 1:4:18:37:40 in chloroform:methanol (9:1, v/v) as described previously [9]. DiI, a fluorescent label that incorporates in the liposome lipid bilayer, was added to the lipid mixture at 0.5 mol% of total lipid. The lipid mixture was dried under reduced nitrogen pressure for 30 min and freeze dried followed by 1 h hydration in HN buffer (10 mM N-2hydroxyethylpiperazine- N-2-ethanesulfonic acid (Hepes), 135 mM NaCl) pH 6.7 and 10 times rapid freezing in liquid N_2_ and thawing in a water bath at 40°C. The so-formed SAINT-O-Somes were extruded 13 times through polycarbonate filters (Costar, Cambridge MA, USA) having a pore size of 50 nm at 40°C, using a high pressure extruder (Lipex Biomembranes Inc., Vancouver, Canada).

Part of the total SAINT-O-Somes preparation was conjugated with a specific antibody (targeted SAINT-O-Somes), the non-modified SAINT-O-Somes (non-targeted) were used as control. Mouse anti-human VCAM-1 (IgG1) was purified from supernatant produced by hybridoma E1/6aa2 as previously described for mouse anti-human E-selectin [9]. The monoclonal VCAM-1 antibody-producing hybridoma E1/6aa2 was kindly provided by Dr. M. Gimbrone Jr. (Harvard Medical School, Boston, MA, USA). The monoclonal mouse anti-human VCAM-1 antibody was thiolated by SATA and was coupled (0.22 mg of antibody/μmol total lipid) to a maleimide group present at the distal end of the PEG chain as previously described [9]. The antibody coupled-SAINT-O-Somes was purified from the free antibody by two rounds of density-gradient ultra-centrifugation each at 242,000 x g at 4°C for 2 h, and followed by overnight dialysis at 4°C against HN buffer of pH 7.4.

For the rapamycin-SAINT-O-Somes preparation, POPC, cholesterol, Mal-PEG-DSPE and SAINT-C-18, were dissolved in chloroform:methanol (9:1, v/v) in a round-bottom flask in a molar ratio of 1.48:1.6:0.2:0.72, respectively. Desired amounts of rapamycin were simultaneously added to the lipid mixture based on the total amount of lipid (20 nmol rapamycin/μmol total lipid) and a drug-lipid film was created using a rotary evaporator and subsequent drying under a nitrogen stream. SAINT-O-Somes were hydrated by adding HN buffer followed by sonication for 10 min and incubation for 1h at room temperature. The extrusion and antibody coupling steps were performed as described for VCAM-1-DiI-SAINT-O-Somes. Free antibody and non-encapsulated rapamycin were removed by passing SAINT-O-Somes through a PD10 column followed by spinning in a Vivaspin centrifugal concentrator (Sartorius AG, Aubagne, France) with a molecular weight cut-off membrane of 300-kDa.

### Characterization of SAINT-O-Somes

The phospholipid phosphate content of the SAINT-O-Somes was determined by phosphate assay [13,14]. The mean particle size was measured by Nicomp^TM^ 380 ZLS particle analyzer. The polydispersity index of the SAINT-O-Somes was determined by dynamic light scattering using a Malvern CGS-3 multiangle goniometer (Malvern Instruments Ltd., Worcestershire, UK) with a JDS Uniphase 22 mWHe–Ne laser operating at 632 nm, an optical fiber-based detector and a digital LV/LSE- 5003 correlator. All measurements were performed at a 90° angle. The zeta-(ζ) potential of the SAINT-O-Somes was determined by laser doppler electrophoresis using a Zetasizer Nano-Z (Malvern Instruments Ltd.). Peterson-Lowry protein assay was performed to determine the amount of antibody coupled to SAINT-O-Somes. SAINT-O-Somes was stored at 4°C under argon.

Rapamycin content of the SAINT-O-Somes was determined by high performance liquid chromatography (Waters Acquity HPLC system, Waters Corporation, Milford, MA, USA) using a Sunfire C18 column, at a flow rate of 1 ml/min and a column temperature of 60°C. The isocratic mobile phase was composed of 70% acetonitrile and 30% water, UV detection was performed at 290 nm.

Size, charge, polydispersity index, the amount of rapamycin encapsulated and the anti-VCAM-1 antibody coupling efficiencies of antiVCAM-1-rapamycin-SAINT-O-Somes are listed in Table 1. The coupling of anti-VCAM-1 antibody to rapamycin-SAINT-O-Somes was determined by Dot-blot as described before [15].

**Table 1:** Characterization of rapamycin-SAINT-O-Somes (*n = 3*)*.

Samples	Size (nm)	Charge (mv)	Polydispersityindex	Rapamycin loading efficiency (%)
Anti-VCAM-1-rapamycin- SAINT-O-Somes	128 ± 4	10.8 ± 0.9	0.06 ± 0.01	73 ± 0.7
Rapamycin- SAINT-O-Somes	122 ± 2	11.0 ± 0.3	0.07 ± 0.01	71 ± 1.2

*values represent averages (± sd) of 3 formulations

### Flow Cytometry

AB8/13 and MPC-5 cells differentiated for 15 days at 37°C were incubated with or without TNFα (10 ng/ml) for 24 h. The cells were detached from the plates by trypsinization and divided over FACS tubes and washed once with ice cold FACS buffer (PBS-containing 5% FCS). Next the cells were incubated with mouse anti-human-VCAM-1 (10 μg/ml) (for AB8/13) and polyclonal rabbit anti-VCAM-1 antibodies (10 μg/ml) (for MPC-5) for 45 min at 4°C followed by three washing steps with ice cold FACS buffer (PBS-containing 5% FCS). Control cells were incubated with mouse anti-rat-IgG2a (Clone OX35, hybridoma supernatant) (5 μg/ml) antibodies (isotype) and quiescent cells were used as controls. Detection was established by incubation with a FITC-tagged rabbit anti-mouse IgG Fab_2_ (Jackson immuno research, PA, USA) (for AB8/13) and alexa_488_-tagged goat-anti-rabbit (Life Technologies) (for MPC-5) secondary antibodies for 30 min at 4°C followed by washing three times with FACS buffer.

Similarly, to determine the association of the anti-VCAM-1-DiI-SAINT-O-Somes, quiescent or TNFα (10 ng/ml; 24 h)-activated AB8/13 cells were incubated with either anti-VCAM-1-DiI-SAINT-O-Somes or DiI-SAINT-O-Somes, all at a concentration of 80 nmol total lipid/ml for 4 h at 37°C. Quiescent cells incubated with an equivalent volume of HN buffer were included as control. The cells were finally resuspended in 0.2 ml FACS buffer and analyzed by flow cytometry (Calibur, BD Biosciences, NJ, USA). The mean fluorescence intensity (MFI) was obtained after analysis with FlowJo^TM^ software package (Tree Star Inc., OR, USA), version 7.6.5). The MFI values of the cells (TNFα-activated and quiescent) incubated with isotype control antibodies were subtracted from test samples. Results presented were determined from the mean of three independent experiments with duplicate incubations per experiment.

### Fluorescence Microscopy

For immunofluorescence detection of nephrin and Tie2, cytospots of primary podocytes were made on a microscope slide. To avoid nonspecific binding of antibodies, blocking with PBS-containing 1% BSA and 5% FCS was performed at room temperature. Guinea pig anti-mouse nephrin (Promega), rat anti-mouse Tie2 (Millipore) and rabbit-IgG (SouthernBiotech, Alabama, USA) (control) antibodies were added and incubated for 1 h at room temperature followed by incubation with respective secondary fluorescent antibodies Goat anti-guinea pig-FITC (SouthernBiotech), goat anti-rat-FITC (SouthernBiotech) and goat anti-rabbit-Alexa_488_ (Life Technologies) for 30 min. The cells were washed three times with PBS after incubation with primary and secondary antibodies. Finally, the nuclei were stained with 4′,6-diamidine-2′-phenylindole dihydrochloride (DAPI) in fluoroshield mounting medium (Abcam, Cambridge, UK). The cells were visualized and analyzed under a fluorescence microscope (DM RXA, Leica Microsystems AG, Wetzlar, Germany), and Leica Q600 Qwin software V01.06, respectively.

For fluorescence microscopy detection of DiI-SAINT-O-Somes, AB8/13 cells (16,000 cells/cm^2^) were seeded in Lab-Tek chamber slides (NUNC, Rochester, NY) and allowed for 15 days to differentiate at 37°C with medium refreshed every 5 days. Cells were activated with TNFα (10 ng/ml) for 24 h followed by incubation with anti-VCAM-1-DiI-SAINT-O-Somes and DiI-SAINT-O-Somes, both at a concentration of 80 nmol total lipid/ml for 4 h at 37°C. 10 min before the end of incubation, the nuclei of the cells were stained with Hoechst 33342 (0.1 mg/ml in serum free medium; Invitrogen) followed by washing three times with ice cold PBS and one final wash with ice cold serum-free culture medium. The chamber was removed and a cover slip was placed on the slide followed by sealing with nail polish. The microscopic images were taken within 1 h.

### Cell Viability Assay

Effects of rapamycin and rapamycin-SAINT-O-Somes on cell viability of AB8/13 cells were determined by Sulforhodamine B (SRB) colorimetric assay [16]. AB8/13 cells (15 days differentiated at 37°C; 5000 cells/cm2) were either activated with TNFα (10 ng/ml) for 24 h or incubated in control medium (non-activated cells). Free rapamycin, rapamycin-SAINT-O-Somes and anti-VCAM-1-rapamycin-SAINT-O-Somes were added to triplicate wells at final concentrations corresponding to 8 or 32 μM rapamycin after which the cells were incubated for an additional 24 h at 37°C. Cells were washed once with PBS and fixed by addition of 10% (wt/vol) Trichloroacetic acid (TCA) at 4°C for 1 hour, followed by staining with 0.4% SRB stain in 1% acetic acid for 30min. Excess non-bound SRB dye was removed by multiple washings with 1% acetic acid after which the cells were dried at room temperature. The protein-bound dye was dissolved in unbuffered 10 mM Tris for 30 min and OD values were measured at 490–550 nm with a Spectrostar microplate reader. The values obtained were normalized versus control cells treated with TNFα.

### Wound Healing Assay

Confluent 6-well culture plate with differentiated AB8/13 cells were incubated in the absence or presence of TNFα (10 ng/ml; 24 h) followed by treatment with free rapamycin, rapamycin-SAINT-O-Somes and anti-VCAM-1-rapamycin-SAINT-O-Somes at a concentration of 10 nmol/ml. After 12 h incubation with the different formulations, a scratch was made with a sterile 200 μl pipette tip followed by a one-time wash with PBS after which cells were refreshed with fresh medium. The wound closure was followed in time for the next 24 h with images taken at 0, 6 and 24 h time points. The extent of wound closure was determined by quantifying cell migration towards the wound using ImageJ.

### Statistical Analysis

Statistical analysis of the results was performed by two-tailed unpaired Student's *t*-test or one- way ANOVA. *p*-values <0.05 were considered to be significant. Data were analyzed with Graphpad prism (Graphpad software 5.0b, San Diego CA, USA).

## Results, Discussion, and Conclusions

To investigate the possibilities of using VCAM-1 as a molecular target to deliver drugs specifically (in)to podocytes, we initiated an *in vitro* study using the human conditionally immortalized podocyte cell line AB8/13 [10]. These cells proliferate at 33°C and differentiate to the podocyte phenotype in 10–15 days at 37°C. In order to establish the differentiation of AB8/13 cells into a podocyte phenotype, mRNA and protein expression of the podocyte-specific markers (Wilms’s Tumor-1 (WT-1) and synaptopodin) were analyzed after 15 days at 37°C. Differentiated AB8/13 cells expressed both WT-1 and synaptopodin, confirming AB8/13 as podocytes (S1 and S2 Figs).

### TNFα Exposure Induces the Expression of VCAM-1 in Differentiated AB8/13 Podocytes

**Fig 1:**
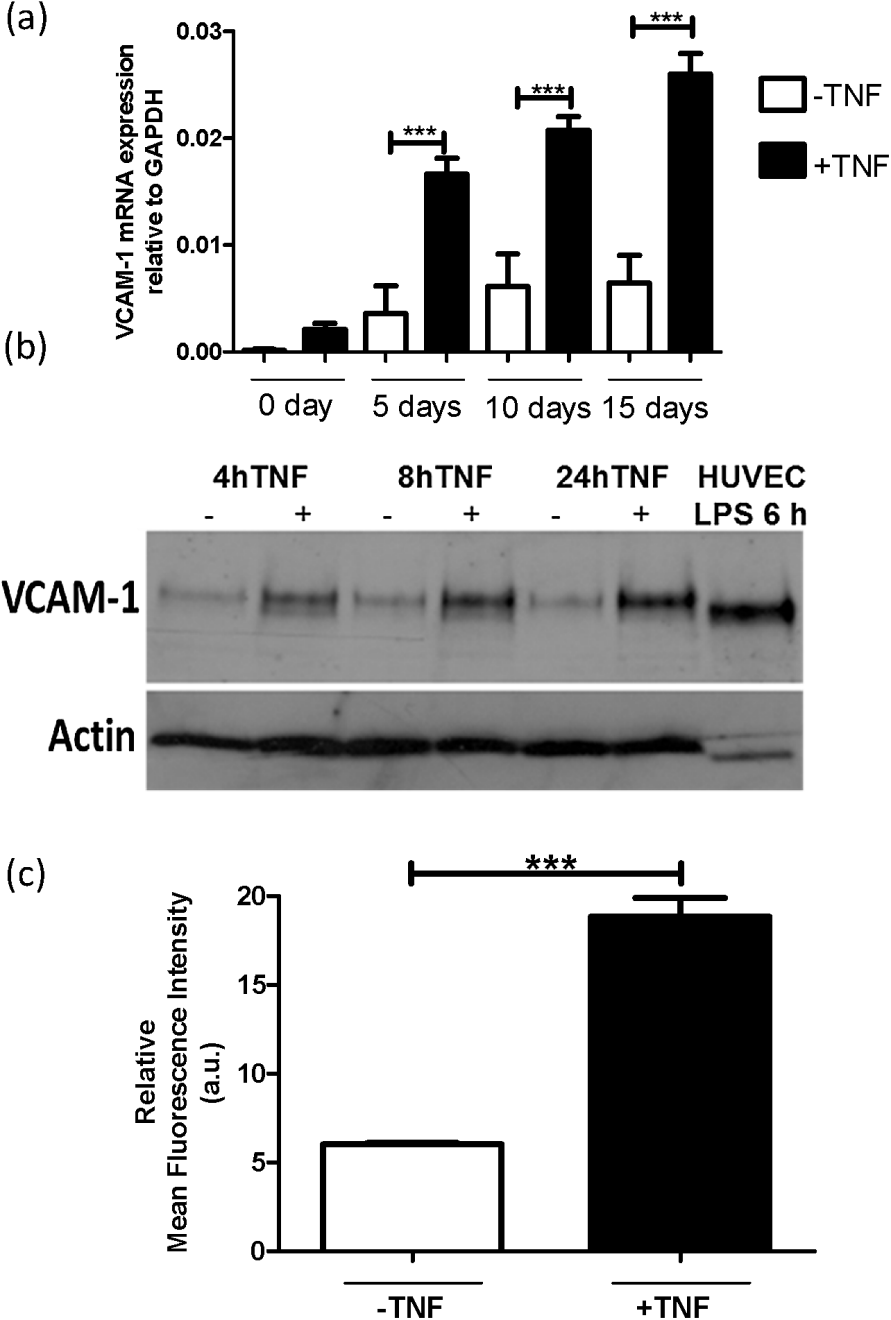
VCAM-1 is expressed in AB8/13 cells and the expression is increased upon TNFα-activation. (**a**) RT-qPCR analysis of human VCAM-1 mRNA expression. The AB8/13 cells were differentiated for 0, 5, 10, and 15 days at 37°C and treated with TNFα (10 ng/ml) for 4 h. (**b**) Western blot analysis of VCAM-1 (~110-kDa) and actin (~42-kDa) (loading control) proteins expression in cell lysate of AB8/13 cells, 15 days differentiated at 37°C in the absence (-) or presence (+) of TNFα for various time points. (**c**) FACS analysis of VCAM-1 protein in TNFα-activated (10 ng/ml; 24 h) and quiescent AB8/13 cells incubated with mouse anti-human-VCAM-1 for 45 min at 4°C followed by incubation with fluorescently-labelled secondary antibody. *p <0.001.

To study the prospects of using adhesion molecule VCAM-1 as a molecular target for podocyte-specific drug delivery purposes, mRNA was isolated from both TNFα (10 ng/ml; 4 h)-activated and quiescent AB8/13 cells up to 15 days of differentiation at 37°C. Gene expression levels of VCAM-1 were compared to the housekeeping gene GAPDH. VCAM-1 was expressed by podocytes and the expression was significantly upregulated by TNFα (Fig 1A). Under the conditions studied, the expression of VCAM-1 gradually increased with the differentiation time of the cells and was at its maximum at 15 days of differentiation (Fig 1A). An approximately 4-fold increase in VCAM-1 mRNA expression was observed in the presence of TNFα when compared to quiescent cells.

Analysis of VCAM-1 protein expression in the differentiated AB8/13 cells corroborated the mRNA data that in the absence of TNFα, there is basal expression of VCAM-1 and that the expression significantly increases in the presence of TNFα (Fig 1B and 1C). To confirm the expression of VCAM-1 protein by podocytes also flow cytometry was performed. Fig 1C shows that anti-VCAM-1 antibodies bound to both activated and quiescent cells. There is a 3-fold higher expression of VCAM-1 in TNFα-activated podocytes compared to quiescent cells (Fig 1C). The binding of VCAM-1 specific antibodies to quiescent cells can be attributed to the basal expression of VCAM-1 in this condition. These results indicate that VCAM-1 can be used as a molecular target to address inflammation activated podocytes using nanocarriers.

### VCAM-1 Is Also Expressed in a Mouse Podocyte Cell Line and Primary Mouse Podocytes

**Fig 2:**
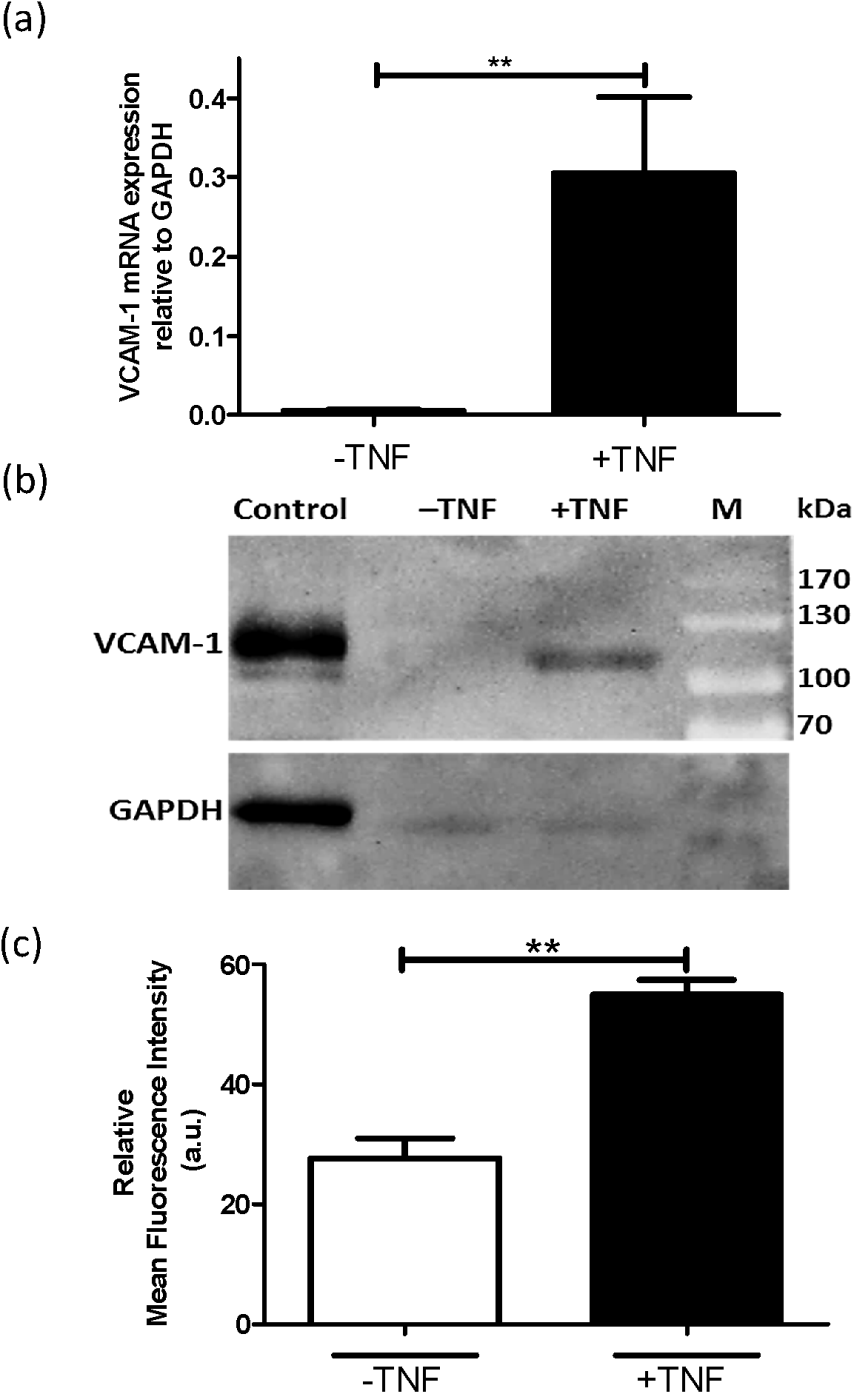
VCAM-1 is expressed by the mouse podocyte cell line MPC-5. (**a**) mRNA expression of VCAM-1 in the absence (-) or presence (+) of TNFα (10 ng/ml; 24 h) as analyzed by RT-qPCR analysis. (**b**) Western blot analysis of protein extracts of MPC-5 cells that were incubated in the absence (-) or presence (+) of TNFα (10 ng/ml; 24 h) prior to harvesting. M and control indicate respectively, pre-stained molecular weight marker and positive control (HUVEC activated with LPS for 6 h). (**c**) FACS analysis of VCAM-1 expression by MPC-5 cells using anti-VCAM-1 antibody by flow cytometry. *p <0.001.

We investigated the expression of VCAM-1 both in a conditionally immortalized mouse podocyte cell line (MPC-5) that was differentiated for 12 days at 37°C and in primary mouse podocytes. TNFα was added to the incubation during the last 24 h before isolation of mRNA or protein. RT-qPCR analysis of podocyte-specific markers synaptopodin and WT-1 showed the expression of both genes confirming MPC-5 cells as podocytes (S3 Fig). Similar to AB8/13 cells, MPC-5 cells expressed VCAM-1 and the expression was significantly upregulated in the presence of TNFα (Fig 2A). Western blot analysis and flow cytometry of MPC-5 cells are in accordance with the gene expression results, showing significant VCAM-1 protein levels in these cells upon activation with TNFα (Fig 2B and 2C).

**Fig 3:**
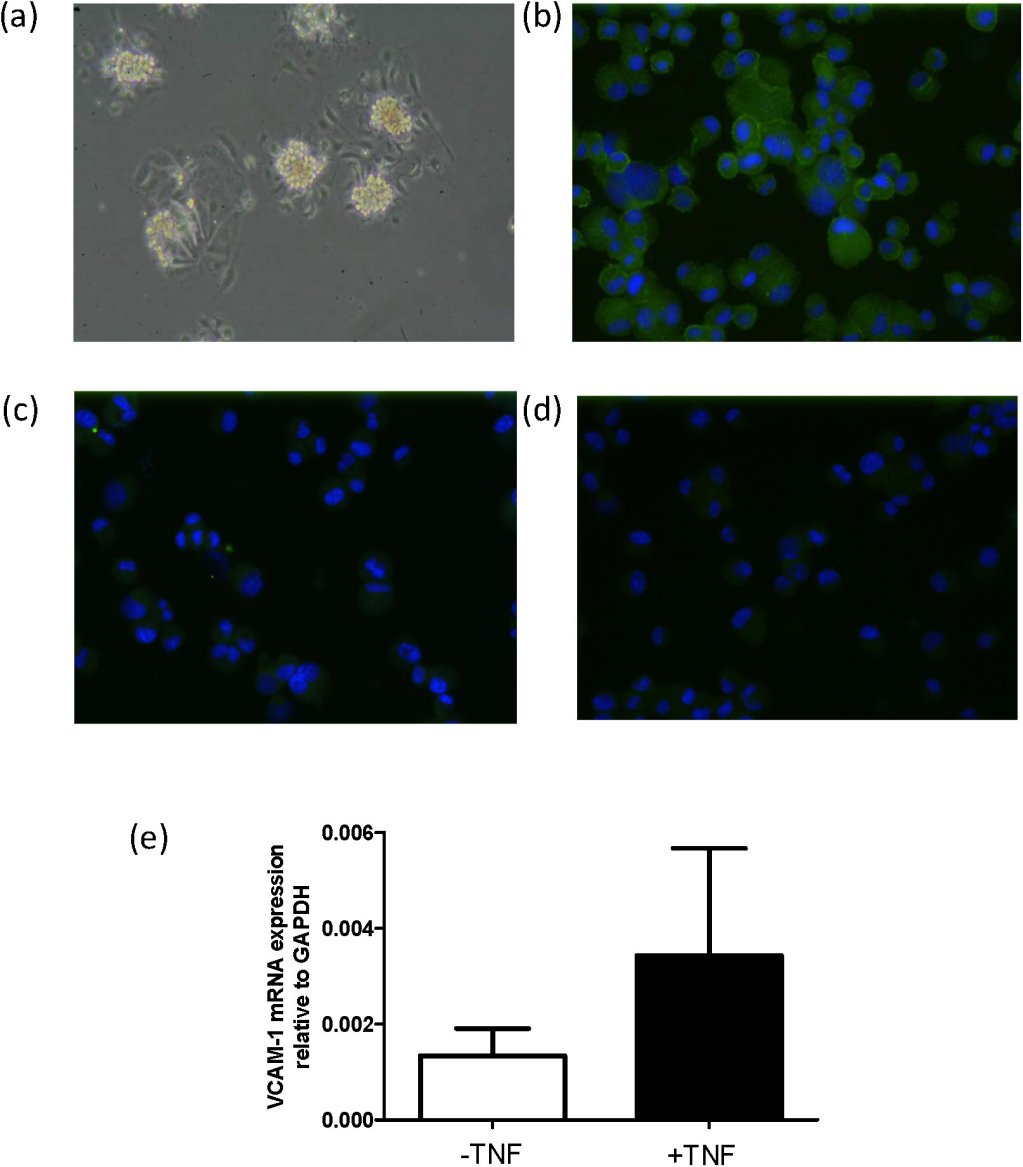
VCAM-1 is expressed in primary mouse podocytes. (**a**) Phase-contrast view of glomerular outgrowths of isolated glomeruli from mouse kidney, 2 days after seeding in podocyte-specific medium. Immuno-staining of cytospots of the ICAM-2 negative fraction after ICAM-2 re-beading, detecting nephrin (**b**), while the endothelial marker Tie2 (**c**) is not detected. Panel (**d**) shows background staining with control rabbit-IgG antibodies (and does not detect anything). Antibodies that specifically bound to the cells are stained in green. Nuclei were stained in blue with DAPI. (**e**) VCAM-1 mRNA expression in mouse primary podocytes in the absence (-) or presence (+) of TNFα, as determined by RT-qPCR. Data are presented as mean values +/- sd, n = 3 from three independent experiments.

Primary mouse glomerular cells grew to confluency within 4–5 days of incubation at 37°C in podocyte-specific medium (Fig 3A), after which the non anti-ICAM-2 beads sorted population was seeded in podocyte-specific medium. Cytospots from these ICAM-2 negative fractions were stained with podocyte and endothelial cell-specific antibodies. Immunofluorescence analysis showed specific detection of the podocyte-specific marker nephrin confirming that the cultured cells had a podocyte-like phenotype (Fig 3B). In contrast, cytospots stained for Tie2 that is restrictedly expressed by endothelial cells showed no fluorescence indicating that there was no contamination with endothelial cells (Fig 3C and 3D). Primary podocytes were either grown in quiescence or activated by TNFα for 6 h and RNA was isolated to determine the expression of podocyte and endothelial markers and VCAM-1. In accordance with the data obtained with AB8/13 cells and MPC-5 cells, VCAM-1 was also expressed in mouse primary podocytes and the expression was upregulated in the presence of TNFα (Fig 3E). RT-qPCR analysis showed the expression of podocyte-specific genes (WT-1 and synaptopodin) confirming these primary cells as podocytes (S4 Fig). There is minute expression of the endothelial-specific genes CD31 and VEcadherin confirming that there is hardly any contamination of glomerular endothelial cells in the primary podocyte culture (S4 Fig). Since the yield of primary podocytes after isolation from mouse kidneys is limited, we only performed low cell number requiring gene expression analysis and immunofluorescence of VCAM-1 in primary podocytes.

Based on these *in vitro* gene and protein expression results in AB8/13, MPC-5, and primary mouse podocytes, we concluded that VCAM-1 is expressed by podocytes and that the expression was significantly upregulated by activation of the cells with the pro-inflammatory cytokine TNFα. This led us to hypothesize that VCAM-1 can be exploited as a molecular target to specifically deliver drugs (in)to podocytes using a nanocarrier system, especially since VCAM-1 receptors were shown to be internalized by cells *via* clathrin-mediated endocytosis [17]. This is a prerequisite for an efficient nanocarrier to successfully deliver its contents inside its target cell [18].

### Association of Anti-VCAM-1-DiI-Saint-O-Somes with Inflammation Activated AB8/13 Podocytes

To deliver therapeutic compounds into podocytes, we used a SAINT-based lipid drug carrier system called SAINT-O-Somes with anti-VCAM-1 antibodies coupled to the distal end of the polyethylene glycol molecules to make them inflammation activated podocyte specific. To investigate the binding and association by the podocytes, SAINT-O-Somes were labeled with DiI in the lipid bilayer. The diameter of DiI-SAINT-O-Somes and anti-VCAM-1-DiI-SAINT-O-Somes was 95+/- 2 and 115+/- 11 nm, respectively. 45+/- 3 of anti-VCAM-1 antibodies were coupled per μmol total lipid of SAINT-O-Somes, which was comparable to earlier studies in which siRNA was incorporated in anti-VCAM-1 antibody harnessed SAINT-O-Somes [8].

**Fig 4:**
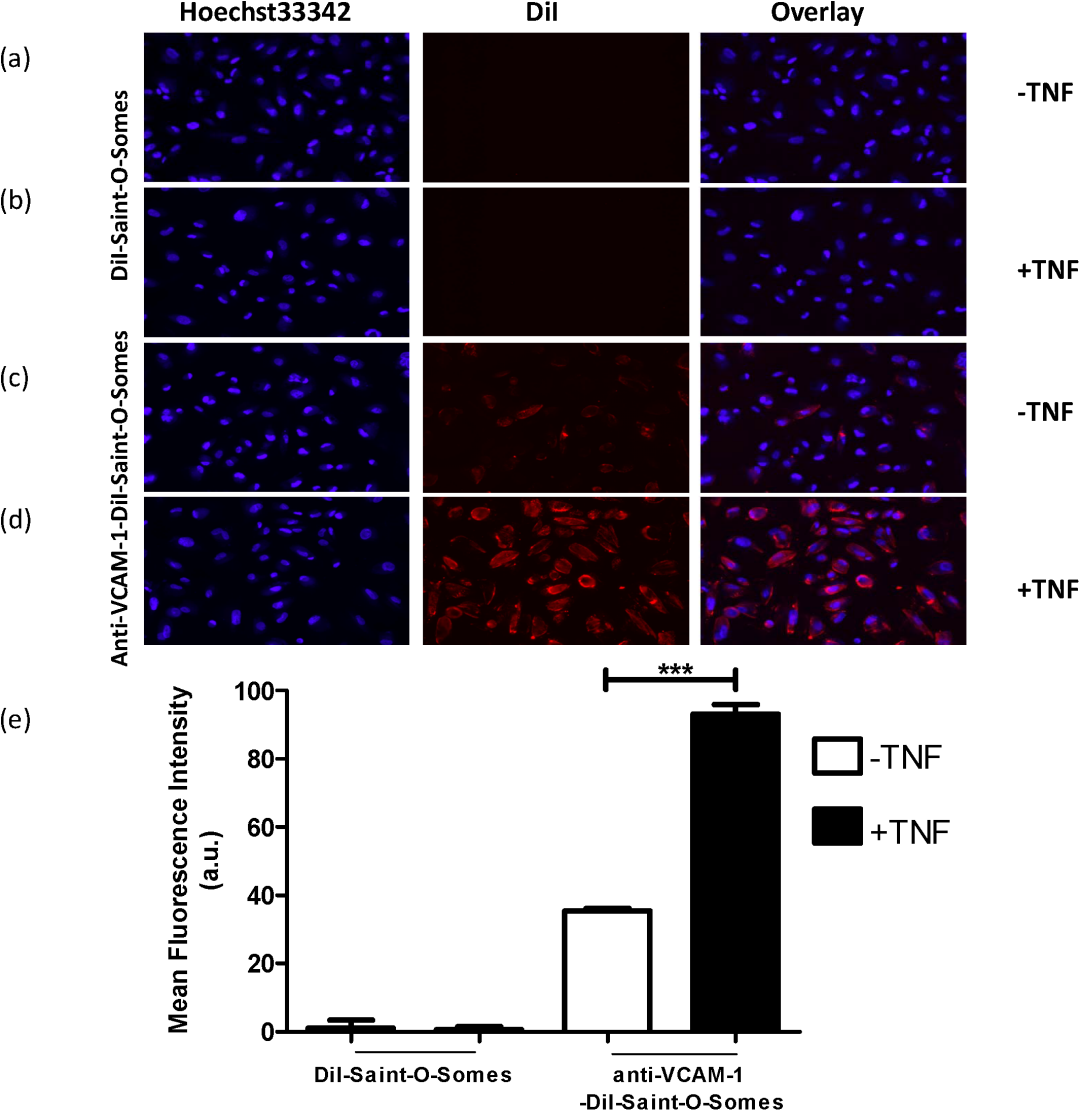
Anti-VCAM-1-DiI-Saint-O-Somes bind to AB8/13 cells. DiI-SAINT-O-Somes (**a**, **b**) and anti-VCAM-1-DiI-SAINT-O-Somes (**c**, **d**) were incubated for 4 h at 37°C with AB8/13 cells in the absence (-) (**A, C**) or presence (+) (**b**, **d**) of TNFα (10 ng/ml; 24 h). The nuclei were stained blue with Hoechst 33342. Original magnification 200x. (**e**) Quantification of binding of anti-VCAM-1-DiI-SAINT-O-Somes or DiI-SAINT-O-Somes to AB8/13 cells. Cells incubated in equal volumes of HN buffer were taken as controls and their values were subtracted from test samples. The mean fluorescence intensity (MFI) was determined by FACS and analyzed by FlowJo^TM^. *p <0.001.

Anti-VCAM-1-DiI-SAINT-O-Somes (targeted) and DiI-SAINT-O-Somes (non-targeted) were incubated for 4 h with quiescent and TNFα-activated, 15 days differentiated AB8/13 cells at 37°C. Fluorescence microscopy analysis showed association of anti-VCAM-1-DiI-SAINT-O-Somes, both in quiescent and TNFα-activated podocytes, with the association with activated cells being much higher (Fig 4C and 4D). In contrast, non-targeted-DiI-SAINT-O-Somes were hardly associated with either quiescent or TNFα-activated cells (Fig 4A and 4B), indicating specificity of targeted-DiI-SAINT-O-Somes for podocytes.

These results were supported by the flow cytometry results, which showed that association of anti-VCAM-1-DiI-SAINT-O-Somes to TNFα-activated podocytes was increased almost 3-fold and more than 150-fold compared to quiescent cells and cells incubated with non-targeted-SAINT-O-Somes, respectively (Fig 4E). The non-targeted-SAINT-O-Somes were hardly associated with the AB8/13 cells, either in the absence or presence of TNFα. These results demonstrate proof-of-concept of targeting podocytes using anti-VCAM-1 nanocarriers.

### Effect of Anti-VCAM-1-rapamycin-SAINT-O-Somes on Inflammation Activated AB8/13 Podocytes

It has been proposed that mTOR is involved in podocyte injury [7]. The delivery of mTOR inhibitor rapamycin to podocytes hence will be an attractive approach to treat podocyte injury. One of the techniques to study rapamycin effects in cells is studying migration of the cells. Rapamycin was encapsulated in the lipid-bilayer of the anti-VCAM-1-SAINT-O-Somes and control SAINT-O-Somes. The coupling of VCAM-1 specific antibody to rapamycin-SAINT-O-Somes was confirmed by Dot-blot assay using anti-VCAM-1 antibody detection of the protein (S5 Fig). The cytotoxicity of rapamycin and anti-VCAM-1-rapamycin-SAINT-O-Somes was analyzed (S6 Fig), demonstrating that incubation of AB8/13 podocytes with free or targeted liposomal rapamycin did only slightly affect cell viability at 8 μM and 32 μM rapamycin.

**Fig 5:**
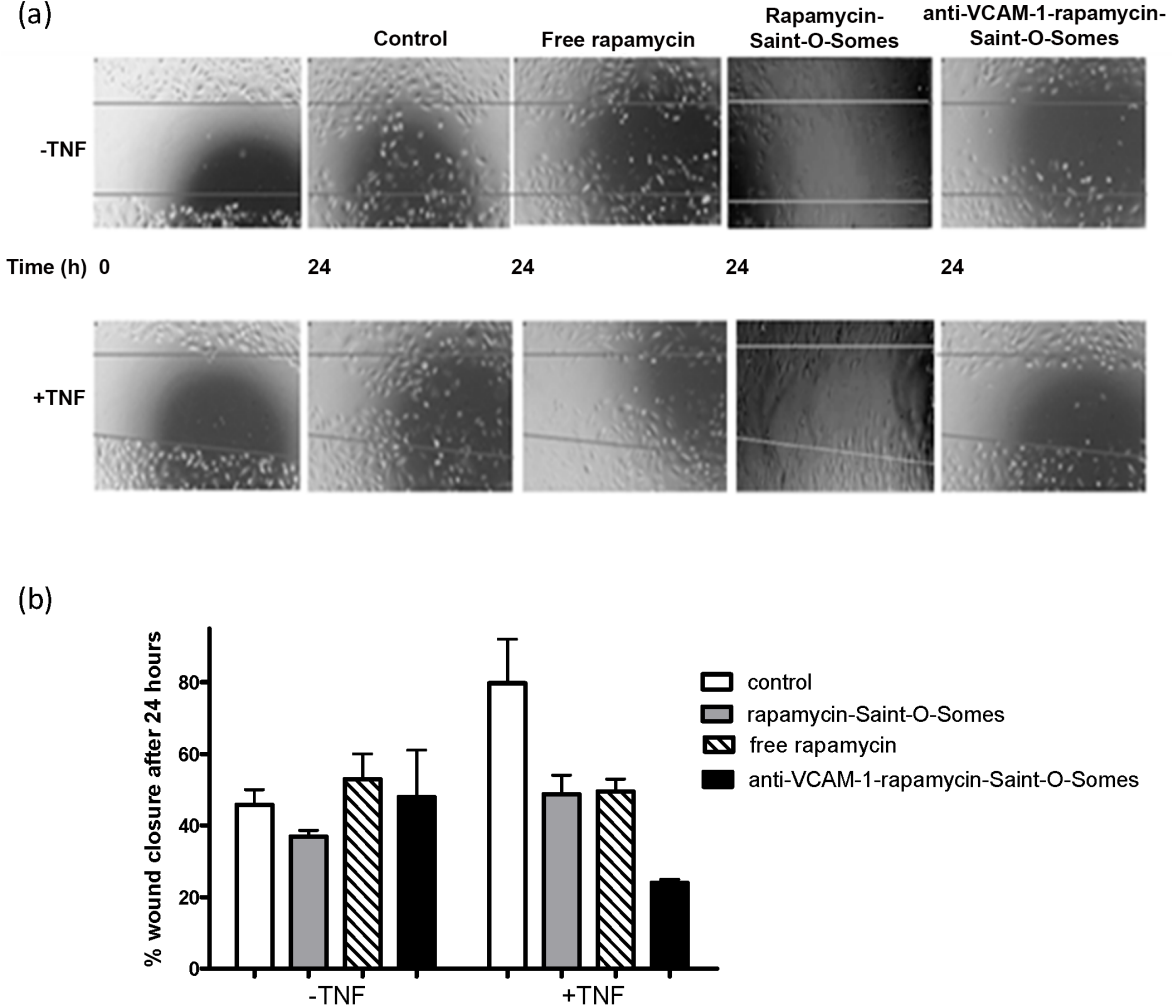
Effect of anti-VCAM-1-rapamycin-SAINT-O-Somes on AB8/13 cells. (**a**) A scratch was made into confluent cell cultures of AB8/13 in the absence (-) or presence (+) of TNFα (10 ng/ml; 24 h). These cells were first treated with HN buffer (control), free drug, rapamycin-SAINT-O-Somes and anti-VCAM-1-rapamycin-SAINT-O-Somes for 12 h, after which a wound was induced and the wound closure was followed in time up to 24 h. (**b**) The number of cells that had migrated into the scratch area (═) were quantified from two independent experiments by ImageJ.

To test the effects of drug loaded anti-VCAM-1-rapamycin-SAINT-O-Somes and compare these with drug loaded non-targeted-SAINT-O-Somes and free drug, a wound healing assay was performed. Light microscopic images from the wound healing assay show that AB8/13 cell migration was almost completely inhibited in TNFα-activated cells treated with anti-VCAM-1-rapamycin-SAINT-O-Somes indicating a cell migration inhibitory effect of rapamycin when applied in the SAINT-O-Somes formulation (Fig 5A). We also observed partial inhibition of cell migration in cells that had not been pretreated with TNFα which is most likely due to the basal expression of VCAM-1 in these cells (Fig 4). Free rapamycin and non-targeted-SAINT-O-Somes loaded with rapamycin showed partial response in TNFα pretreated cells, but these effects were less pronounced as compared to the anti-VCAM-1-rapamycin-SAINT-O-Somes (Fig 5A). Quantification of closure of the wound area showed a 3-fold inhibition of the wound closure in 24 h by the targeted formulation when compared to control cells (Fig 5B). The observed cell migration inhibitory effects are likely due to the inhibition of the actin cytoskeleton by rapamycin [19].

Together these experiments provide conceptual proof that VCAM-1 can be employed as a molecular target to deliver therapeutic compounds into injured podocytes. As mentioned earlier, the fenestrations of the glomerular barrier widen during podocytopathies enabling the nano-sized carriers such as liposomes to reach injured podocytes. In glomerular diseases, not only podocytes but rather all GFB cells, kidney arterioles and venules are injured leading to the expression of various adhesion molecules including VCAM-1. Earlier studies demonstrate that VCAM-1 expression is upregulated in vascular endothelial cells under inflammatory conditions [8,20,21]. Hence, one can expect that rapamycin loaded anti-VCAM-1-SAINT-O-Somes will be internalized by inflammation triggered endothelial cells, similar to previously reported siRNA loaded anti-VCAM-1-SAINT-O-Somes [8, 22]. The expression of VCAM-1 on (subsets of) endothelial cells and on activated podocytes has obviously implications for targeted drug delivery. Although one would like to differentiate between uptake in endothelial cell and podocytes from a scientific proof-of-concept point-of-view, such an exploration in animal models may pose several technical hurdles. First, renal function should largely be preserved in the early stages of the experimental animal model which infers that only a subset of glomeruli (and also the podocytes and endothelial cells) will be disease affected. Podocyte- and endothelium-associated expression of VCAM-1 will furthermore strongly depend on the progression of renal disease in the experimental model. We have earlier demonstrated that the expression of VCAM-1 in glomerular endothelial cells is low in experimental glomerulonephritis, likely because of high microRNA 126 levels which have been shown to down regulate VCAM-1 protein expression [23]. Podocytes and endothelial cell only constitute a small percentage of the total kidney mass, and accumulation of anti-VCAM-1-SAINT-O-Somes in these respective cell types can be demonstrated by immunohistochemical staining. This strategy proved successful for demonstrating in vivo glomerular endothelial cell targeting by anti-E-selectin liposomes [24]. Identification of the cell type involved in glomerular accumulation of the nanocarriers can be done by double staining with podocyte- or endothelium-specific cell markers to get answers on which cell type actually has accumulated the immunoliposomes. Moreover, since both these cell types play an important role in inflammatory processes in the kidney we hypothesize that drug delivery to these cell types may lead to additive therapeutic effects, providing that the delivered drug has beneficial effects in both cell types. Rapamycin may be a good candidate for such a nanocarriers-based approach. We presume that an anti-VCAM-1-coated nanocarrier could be an option to therapeutically address more than one cell type under inflammatory conditions in kidney diseases. Establishing podocyte targeting *in vivo* in a relevant disease model is an obvious and interesting perspective for future research.

## Supporting Information

S1 Fig Wilm’s Tumor-1 (WT-1) is expressed by AB8/13 cells. AB8/13 cells were allowed to differentiate for 15 days at 37°C and RNA was isolated from cells incubated in the absence or presence of TNFα (10 ng/ml, 24 h). WT-1 mRNA expression relative to GAPDH was analyzed by RT-qPCR. Data are presented as mean values +/- sd, n = 3 from three independent experiments.
(TIF)

S2 Fig AB8/13 cells express the podocyte-specific marker synaptopodin. Synaptopodin detection on a Western blot loaded with cell lysates of AB8/13 cells, 15 days differentiated at 37°C in the absence (-) or presence(+) of TNFα for 24 h (10 ng/ml), respectively. The molecular mass (kDa) of pre-stained marker (M) is indicated at the left hand side of the blot. 6 h LPS-activated HUVEC cell lysate and mouse kidney lysate were used as negative and positive controls, respectively. Arrows indicate the bands of synaptopodin and GAPDH as loading control.
(TIF)

S3 Fig Mouse podocyte cell line (MPC-5) expresses podocyte markers. mRNA expression of mouse podocyte-specific markers synaptopodin and WT-1 in the absence (-) or presence (+) of TNFα (10 ng/ml; 24 h) relative to mouse GAPDH, as analyzed by RT-qPCR analysis. Data are presented as mean values +/- sd, n = 3 from three independent experiments.
(TIF)

S4 Fig WT-1 and synaptopodin expression by primary mouse podocytes. RNA was isolated from the ICAM-2 negative glomerular cell fraction and analyzed for the mRNA expression of podocyte (WT-1 and synaptopodin) and endothelial (CD31 and VEcadherin) cell-specific markers, relative to mouse GAPDH, using RT-qPCR. Data are presented as mean values +/- sd, n = 3 from three independent isolates.
(TIF)

S5 Fig Dot-blot assay to demonstrate anti-VCAM-1 antibody conjugation to rapamycin-SAINT-O-Somes. Anti-VCAM-1-rapamycin- SAINT-O-Somes and rapamycin-SAINT-O-Somes were loaded in dilutions ranging from 10x-80x. The successful coupling of anti-VCAM-1 antibody to rapamycin-SAINT-O-Somes was confirmed using fluorescent secondary antibody detecting the anti-VCAM-1 antibody (green). Rapamycin-SAINT-O-Somes without anti-VCAM-1 antibody conjugated did not yield a signal.
(TIF)

S6 Fig Effect of rapamycin on viability of AB8/13 cells. Cell viability of AB8/13 podocytes,15 days differentiated at 37°C, incubated with rapamycin formulations for 24h. Cell viability was assessed by SRB staining and normalized to TNFα treated control cells. Data shown are mean±SE (n = 3 for drug treated cells and n = 48 for controls). * p<0.05 versus resting or activated control podocytes.
(TIF)

## References

[1] Leeuwis JW, Nguyen TQ, Dendooven A, Kok RJ, Goldschmeding R (2010) Targeting podocyte-associated diseases. Adv Drug Deliv Rev 62: 1325–1336. 10.1016/j.addr.2010.08.012 20828590

[2] Siddiqi FS, Advani A (2013) Endothelial-podocyte crosstalk: the missing link between endothelial dysfunction and albuminuria in diabetes. Diabetes 62: 3647–3655. 10.2337/db13-0795 24158990 PMC3806598

[3] Haraldsson B, Jeansson M (2009) Glomerular filtration barrier. Curr Opin Nephrol Hypertens 18: 331–335. 10.1097/MNH.0b013e32832c9dba 19458528

[4] Chiang WC, Geel TM, Altintas MM, Sever S, Ruiters MH, Reiser J (2010) Establishment of protein delivery systems targeting podocytes. PLoS ONE 5: e11837 10.1371/journal.pone.0011837 20686602 PMC2912276

[5] Hauser PV, Pippin JW, Kaiser C, Krofft RD, Brinkkoetter PT, Hudkins KL, et al (2010) Novel siRNA delivery system to target podocytes in vivo. PLoS ONE 5: e9463 10.1371/journal.pone.0009463 20209128 PMC2830889

[6] Canaud G, Bienaimé F, Viau A, Treins C, Baron W, Nguyen C, et al (2013) AKT2 is essential to maintain podocyte viability and function during chronic kidney disease. Nat Med 19: 1288–1296. 10.1038/nm.3313 24056770

[7] Fogo AB (2011) The targeted podocyte. J Clin Invest 121: 2142–2145. 10.1172/JCI57935 21606599 PMC3104780

[8] Kowalski PS, Lintermans LL, Morselt HW, Leus NG, Ruiters MH, Molema G, et al (2013) Anti-VCAM-1 and anti-E-selectin SAINT-O-Somes for selective delivery of siRNA into inflammation-activated primary endothelial cells. Mol Pharm 10: 3033–3044. 10.1021/mp4001124 23819446

[9] Adrian JE, Morselt HWM, Süss R, Barnert S, Kok JW, Asgeirsdóttir SA, et al (2010) Targeted SAINT-O-Somes for improved intracellular delivery of siRNA and cytotoxic drugs into endothelial cells. J Control Release 144: 341–349. 10.1016/j.jconrel.2010.03.003 20226822

[10] Saleem MA, O’Hare MJ, Reiser J, Coward RJ, Inward CD, Farren T, et al (2002) A conditionally immortalized human podocyte cell line demonstrating nephrin and podocin expression. J Am Soc Nephrol 13: 630–638. 11856766 10.1681/ASN.V133630

[11] Mundel P, Reiser J, Zúñiga Mejía Borja A, Pavenstädt H, Davidson GR, Kriz W, et al (1997) Rearrangements of the cytoskeleton and cell contacts induce process formation during differentiation of conditionally immortalized mouse podocyte cell lines. Exp Cell Res 236: 248–258. 9344605 10.1006/excr.1997.3739

[12] Jin E, Liu J, Suehiro J, Yuan L, Okada Y, Nikolova-Krstevski V, et al (2009) Differential roles for ETS, CREB, and EGR binding sites in mediating VEGF receptor 1 expression in vivo. Blood 114: 5557–5566. 10.1182/blood-2009-05-220434 19822898 PMC2798866

[13] Böttcher CJF, Van gent CM (1961) A rapid and sensitive sub-micro phosphorus determination. Analytica Chimica Acta 24: 203–204.

[14] Rouser G, Fkeischer S, Yamamoto A (1970) Two dimensional then layer chromatographic separation of polar lipids and determination of phospholipids by phosphorus analysis of spots. Lipids 5: 494–496. 5483450 10.1007/BF02531316

[15] Khattree N, Ritter LM, Goldberg AFX (2013) Membrane curvature generation by a C-terminal amphipathic helix in peripherin-2/rds, a tetraspanin required for photoreceptor sensory cilium morphogenesis. J Cell Sci 126: 4659–4670. 10.1242/jcs.126888 23886945 PMC3795338

[16] Vichai V, Kirtikara K (2006) Sulforhodamine B colorimetric assay for cytotoxicity screening. Nat Protocols 1: 1112–1116. 17406391 10.1038/nprot.2006.179

[17] Ricard I, Payet MD, Dupuis G (1998) VCAM-1 is internalized by a clathrin-related pathway in human endothelial cells but its alpha 4 beta 1 integrin counter-receptor remains associated with the plasma membrane in human T lymphocytes. Eur J Immunol 28: 1708–1718. 9603478 10.1002/(SICI)1521-4141(199805)28:05<1708::AID-IMMU1708>3.0.CO;2-Y

[18] Sapra P, Allen TM (2002) Internalizing antibodies are necessary for improved therapeutic efficacy of antibody-targeted liposomal drugs. Cancer Res 62: 7190–7194. 12499256

[19] Vollenbröker B, George B, Wolfgart M, Saleem MA, Pavenstädt H, Weide T (2009) mTOR regulates expression of slit diaphragm proteins and cytoskeleton structure in podocytes. Am J Physiol Renal Physiol 296: F418–F426. 10.1152/ajprenal.90319.2008 19019920

[20] Kułdo JM, Ásgeirsdóttir SA, Zwiers PJ, Bellu AR, Rots MG, Schalk JA, et al (2013) Targeted adenovirus mediated inhibition of NF-κB-dependent inflammatory gene expression in endothelial cells in vitro and in vivo. J Control Release 166: 57–65. 10.1016/j.jconrel.2012.12.016 23266453

[21] Gao H-X, Campbell SR, Burkly LC, Jakubowski A, Jarchum I, Banas B, et al (2009) TNF-like weak inducer of apoptosis (TWEAK) induces inflammatory and proliferative effects in human kidney cells. Cytokine 46: 24–35. 10.1016/j.cyto.2008.12.001 19233685

[22] Kowalski PS, Zwiers PJ, Morselt HW, Kuldo JM, Leus NG, Ruiters MH, et al (2014) Anti-VCAM-1 SAINT-O-Somes enable endothelial-specific delivery of siRNA and downregulation of inflammatory genes in activated endothelium in vivo. J. Control. Rel. 176: 64–75. 10.1016/j.jconrel.2013.12.029 24389338

[23] Asgeirsdóttir SA, van Solingen C, Kurniati NF, Zwiers PJ, Heeringa P, van Meurs M, et al (2012) MicroRNA-126 contributes to renal microvascular heterogeneity of VCAM-1 protein expression in acute inflammation. Am J Physiol Renal Physiol 302: F1630–F1639. 10.1152/ajprenal.00400.2011 22419694

[24] Ásgeirsdóttir SA, Zwiers PJ, Morselt HWM, Moorlag HE, Bakker HI, Heeringa P, et al (2008) Inhibition of proinflammatory genes in anti-GBM glomerulonephritis by targeted dexamethasone-loaded AbEsel liposomes. Am. J. Physiol.: Renal Physiol 294: F554–F561. 10.1152/ajprenal.00391.2007 18160627

