# Supplementary figures and images for: Targeting Rapamycin to Podocytes Using a Vascular Cell Adhesion Molecule-1 (VCAM-1)-Harnessed SAINT-Based Lipid Carrier System
Source: PLoS One. 2015 Sep 25;10(9):e0138870. doi: 10.1371/journal.pone.0138870 (PMC4583306; doi:10.1371/journal.pone.0138870)

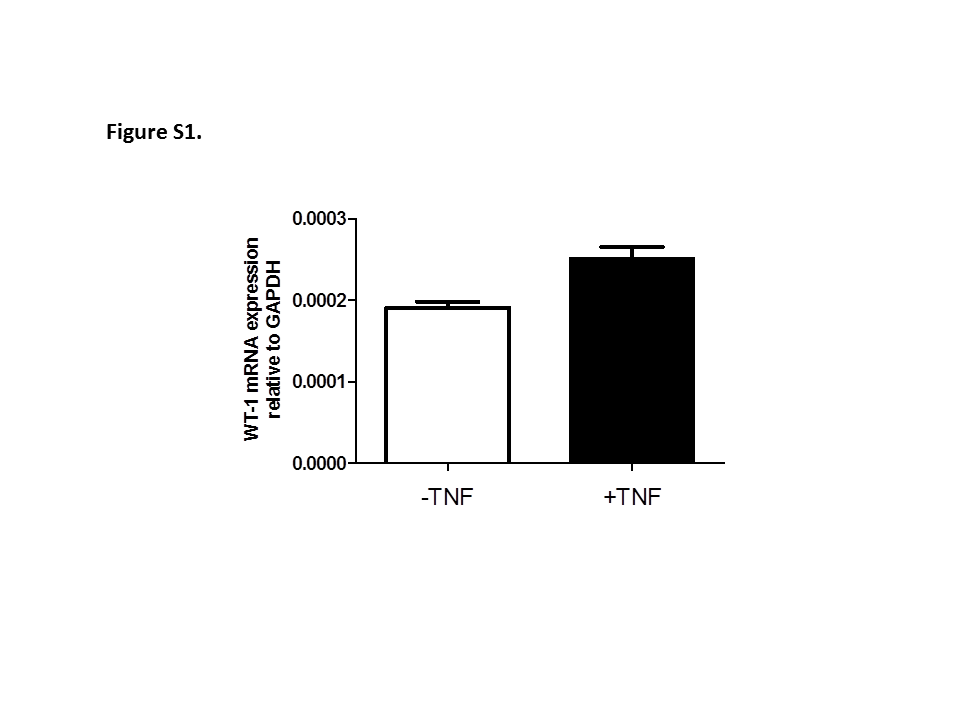

Supplement: S1 Fig — AB8/13 cells were allowed to differentiate for 15 days at 37°C and RNA was isolated from cells incubated in the absence or presence of TNFα (10 ng/ml, 24 h). WT-1 mRNA expression relative to GAPDH was analyzed by RT-qPCR. Data are presented as mean values +/- sd, n = 3 from three independent experiments. (TIF) [file pone.0138870.s001.tif]

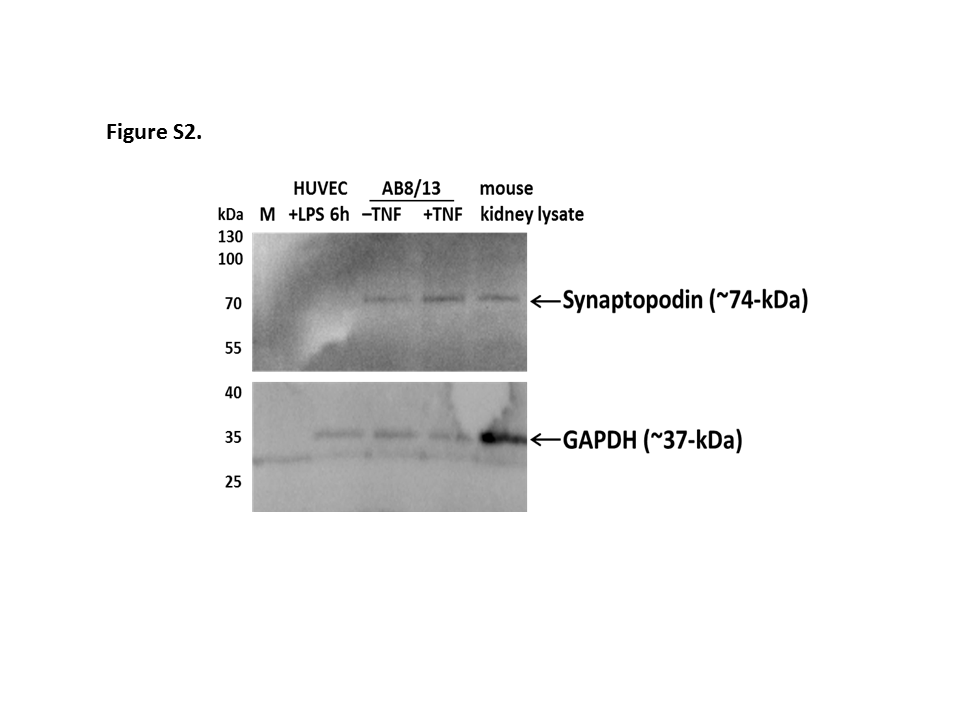

Supplement: S2 Fig — Synaptopodin detection on a Western blot loaded with cell lysates of AB8/13 cells, 15 days differentiated at 37°C in the absence (-) or presence(+) of TNFα for 24 h (10 ng/ml), respectively. The molecular mass (kDa) of pre-stained marker (M) is indicated at the left hand side of the blot. 6 h LPS-activated HUVEC cell lysate and mouse kidney lysate were used as negative and positive controls, respectively. Arrows indicate the bands of synaptopodin and GAPDH as loading control. (TIF) [file pone.0138870.s002.tif]

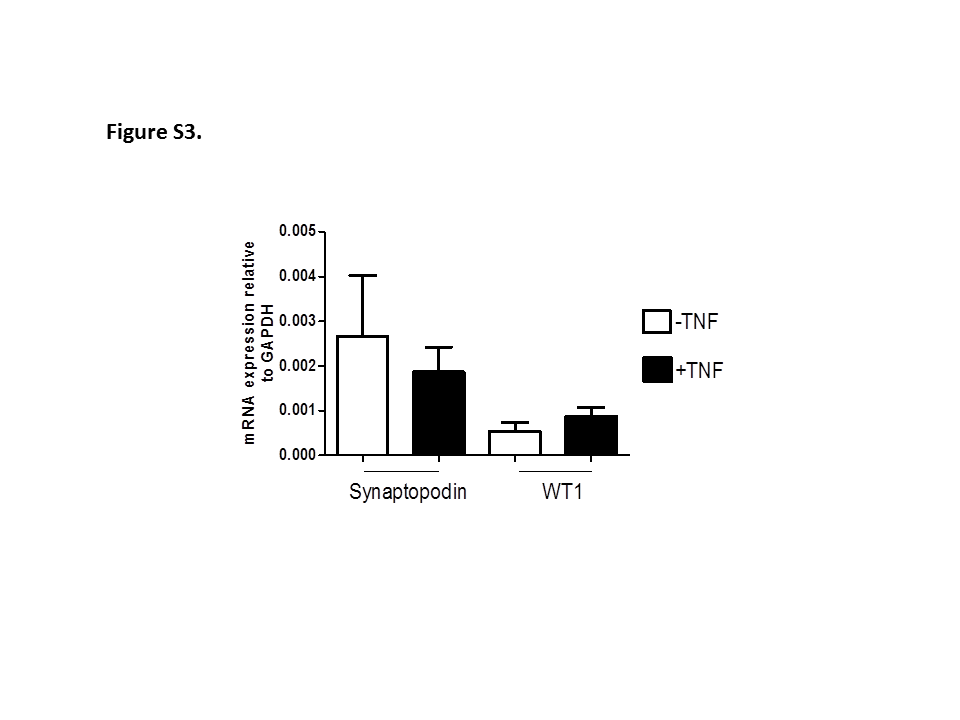

Supplement: S3 Fig — mRNA expression of mouse podocyte-specific markers synaptopodin and WT-1 in the absence (-) or presence (+) of TNFα (10 ng/ml; 24 h) relative to mouse GAPDH, as analyzed by RT-qPCR analysis. Data are presented as mean values +/- sd, n = 3 from three independent experiments. (TIF) [file pone.0138870.s003.tif]

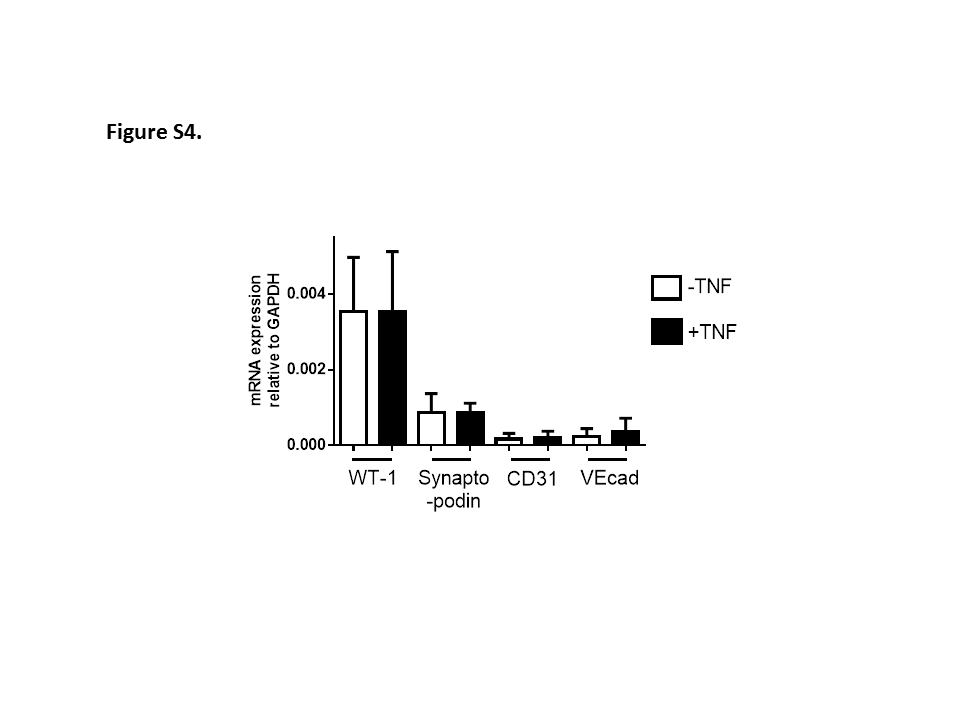

Supplement: S4 Fig — RNA was isolated from the ICAM-2 negative glomerular cell fraction and analyzed for the mRNA expression of podocyte (WT-1 and synaptopodin) and endothelial (CD31 and VEcadherin) cell-specific markers, relative to mouse GAPDH, using RT-qPCR. Data are presented as mean values +/- sd, n = 3 from three independent isolates. (TIF) [file pone.0138870.s004.tif]

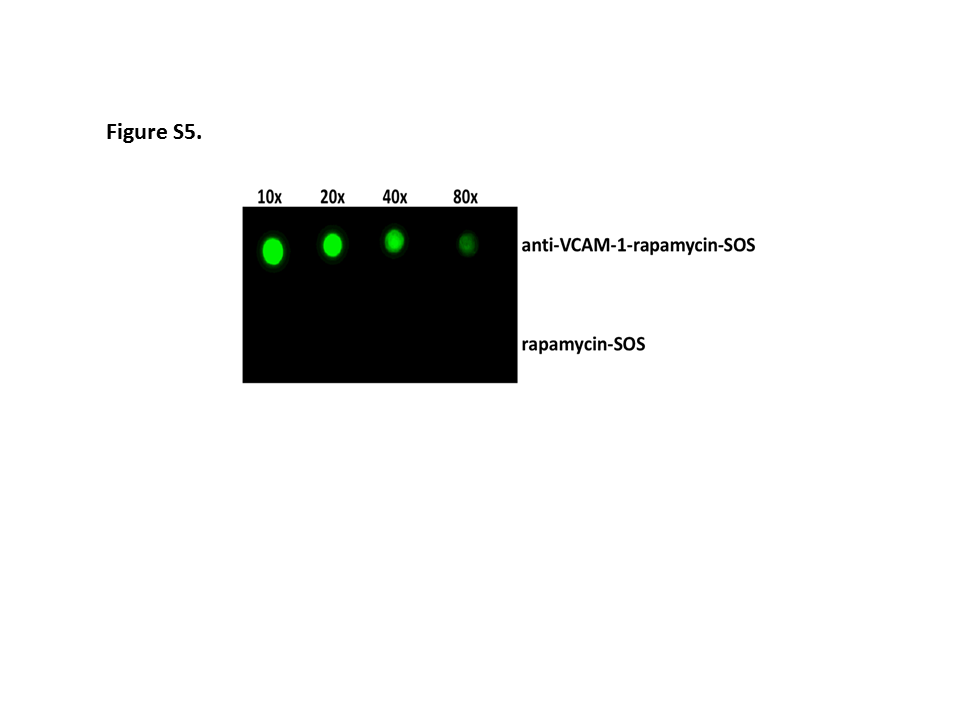

Supplement: S5 Fig — Anti-VCAM-1-rapamycin- SAINT-O-Somes and rapamycin-SAINT-O-Somes were loaded in dilutions ranging from 10x-80x. The successful coupling of anti-VCAM-1 antibody to rapamycin-SAINT-O-Somes was confirmed using fluorescent secondary antibody detecting the anti-VCAM-1 antibody (green). Rapamycin-SAINT-O-Somes without anti-VCAM-1 antibody conjugated did not yield a signal. (TIF) [file pone.0138870.s005.tif]

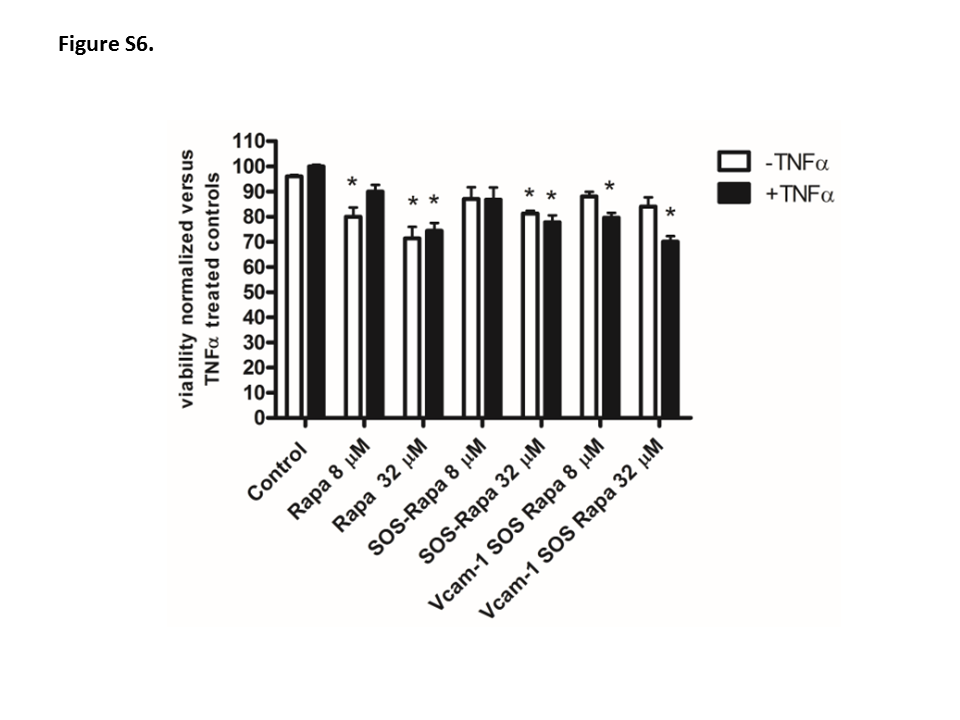

Supplement: S6 Fig — Cell viability of AB8/13 podocytes,15 days differentiated at 37°C, incubated with rapamycin formulations for 24h. Cell viability was assessed by SRB staining and normalized to TNFα treated control cells. Data shown are mean±SE (n = 3 for drug treated cells and n = 48 for controls). * p<0.05 versus resting or activated control podocytes. (TIF) [file pone.0138870.s006.tif]
